# Predictable bonding of adhesive indirect restorations: factors for success

**DOI:** 10.1038/s41415-021-3336-x

**Published:** 2021-09-10

**Authors:** Christopher O´Connor, Dimitrios Gavriil

**Affiliations:** School of Dental Sciences, Restorative Department, Newcastle University, Framlington Place, Newcastle upon Tyne, Tyne & Wear, NE2 4BW, UK

## Abstract

Adhesive indirect restorations are a popular restorative treatment option. This article discusses the many factors that contribute to their successful adhesive cementation, including a review of how to surface treat and manage contaminants across the wide range of indirect materials available.

## Key points

- Critiques the current various adhesive strategies used by resin cements at the tooth tissue interface.
- Describes how to prepare the fitting surface of a wide range of restorative materials for adhesive cementation.
- Discusses how to mitigate against contaminants during the adhesive cementation process.

## Introduction

The adhesive technology required to bond indirect restorations to tooth structure was first introduced in the 1980s.^1^^,^^2^ Adhesive indirect restorations have since become a mainstay of restorative dentistry. Their advantages are twofold: firstly, they allow for a simplified and often more conservative preparation design; and secondly, they confer additional strength to the bonded restoration.^3^

**Fig. 1:**
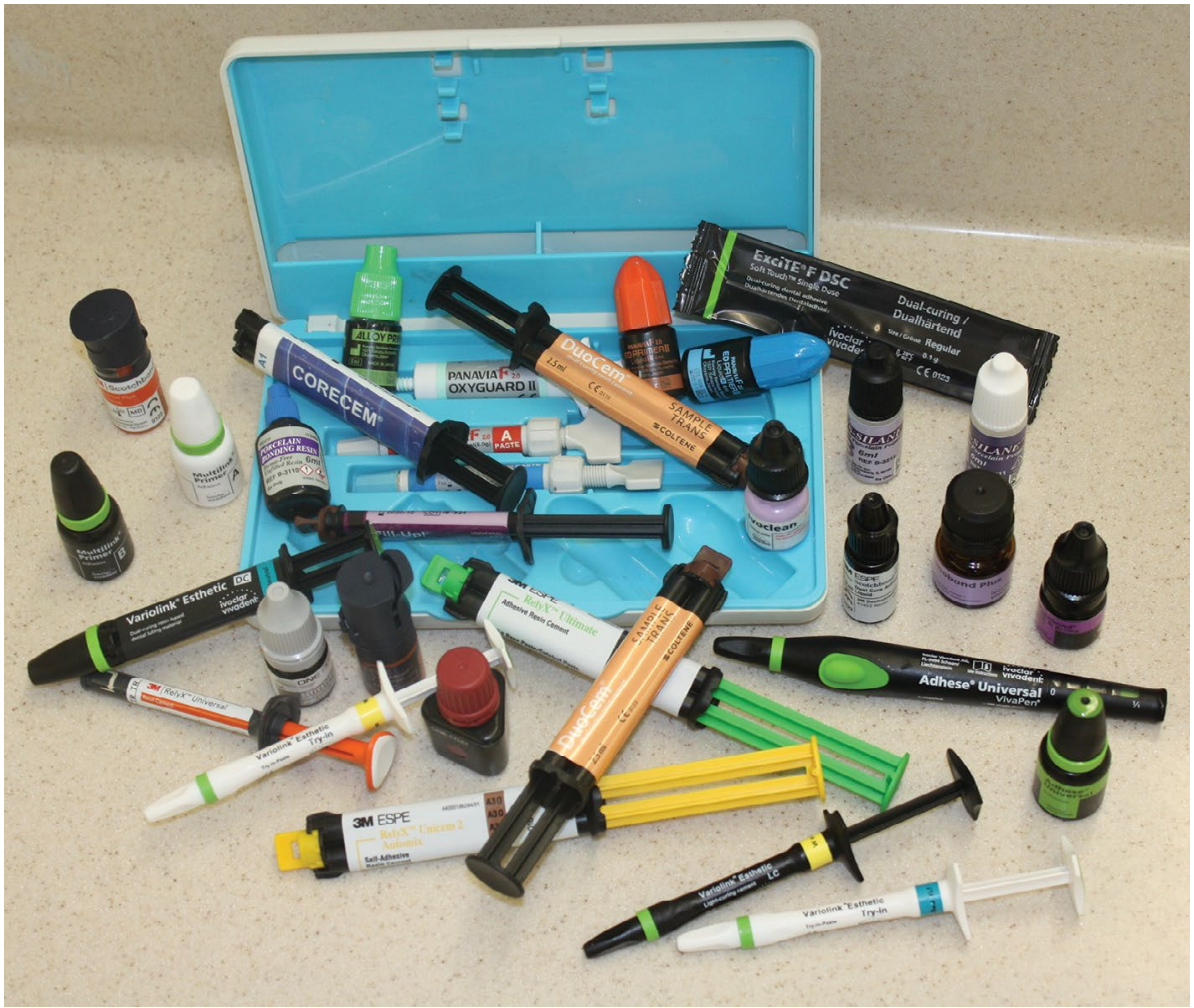
A small sample of the many adhesive cementation systems available for indirect restorations

Implementing the correct bonding strategy is critical to the predictability of indirect restorations but understanding how to condition the fitting surface, across the wide range of material options available, can become confusing (Fig. 1). This article provides an evidence-based summary of the factors dentists should consider when bonding adhesive indirect restorations.

## Planning an adhesive indirect restoration

**Fig. 2:**
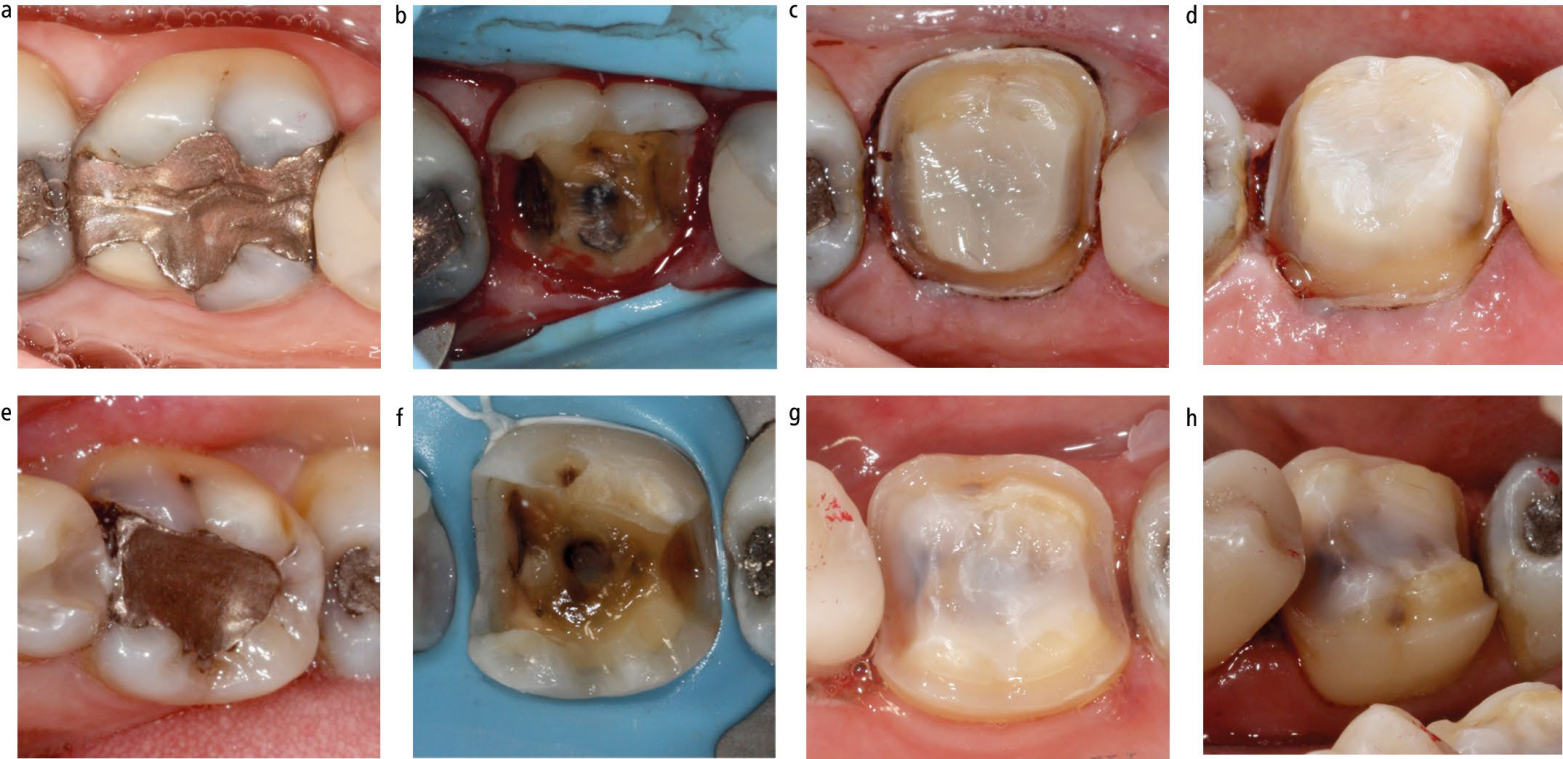
Two contrasting approaches. a, b, c, d) Images show a tooth that was prepared for a passively cemented cuspal coverage restoration (c, d) because a significant proportion of the cavity margin was subgingival and into dentine (b). e, f, g, h) Images show a tooth that was prepared for an adhesively cemented cuspal coverage restoration because the cavity margins were supragingival and within enamel (f)

Not all indirect restorations require adhesive cementation and there is often merit in planning a restoration compatible with a passive cementation strategy (Fig. 2). Passively cemented restorations are more retrievable, less technique-sensitive and can be preferred in clinical situations where adhesive cementation becomes unpredictable.

Passive cementation can be used when a tooth has been prepared to meet specific geometric requirements to confer resistance and retention form.^4^ These geometric requirements are not required for adhesive restorations, but while it is theoretically possible to bond a restoration to an entirely flat surface (relying on adhesive technology alone), clinicians often choose to incorporate some retentive features in their preparations to reduce the stress on the bond interface.

In general, adhesive cementation can be considered when:

- Moisture control of the subject tooth can be assured during the cementation process,^5^ ideally with a rubber dam
- The substrate of the subject tooth is suitable for bonding (ideally with an enamel peripheral rim)^6^
- The procedure can meet patients' requirements; for example, cost, aesthetics, length of procedure.

Having an appropriate material selection and compatible preparation design is a key factor to ensure the success of an adhesive restoration. The success of any adhesive restoration may also be influenced by a number of other factors (for example, oral hygiene, diet and harmony with the occlusal scheme); however, a more thorough analysis is beyond the scope of this article.

## Overview of current adhesive strategies for adhesive cements

There are a variety of adhesive strategies to achieve bonding at the interface between tooth tissues and resin cements (Table 1).

**Table 1:** Advantages and disadvantages of current adhesive strategies

Adhesive strategies	Advantages	Disadvantages
Etch-and-rinse (three-step or two-step)	Gold standard for bonding to enamel^17^ No requirement for technique-sensitive selective etching	Technique-sensitive bonding to dentine^18^ Two-step systems prone to hydrolytic degradation^19^
Self-etch (two-step or one-step)	Chemical bonding to dentine possible via functional monomers^20^ Less influenced by dentine moisture^21^	Lower enamel bond strength, especially to uncut (aprismatic) enamel^22^ One-step systems prone to hydrolytic degradation^8^
Universal (multi-mode)	Less technique-sensitive; can be used effectively either in etch-and-rinse, self-etch or selective enamel etching mode Can be used to prime restoration fitting surface if they include functional adhesive monomers (for example, MDP, silane)^23^	Combination of hydrophilic/hydrophobic monomers makes them susceptible to hydrolytic degradation^24^
Self-adhesive	Ease of use (do not require pre-treatment of tooth surface with etching solutions or bonding agents)^9^	Lower bond strength to enamel and dentine compared to conventional resin cements^10^^,^^11^
Immediate dentine sealing	Reduced risk of dentine sensitivity^25^ Increased bond strength to exposed dentine^15^^,^^26^ Allows for simultaneous blocking out of undercuts in preparations for indirect restorations^12^	Intraoral APA normally required at cementation visit^16^ Resin-based provisionals can be hard to retrieve and require separating medium^12^ Currently only evidence for use with light-cure adhesive resin^12^^,^^16^ which may limit use for thick/opaque restorations

Evidence suggests that bonding systems containing a distinct primer (hydrophilic) and adhesive (hydrophobic) step, such as three-step etch-and-rinse and two-step self-etch, are advantageous in reducing water sorption and hydrolytic degradation of the bond, especially when the exposed margin is into dentine.^7^ Despite this evidence, manufacturers are keen to simplify the bonding process with one-bottle combined adhesive systems. Clinicians should be mindful of this simplification because it often comes at the relative detriment of the bonding efficacy.^8^

A relatively recent development is the introduction of self-adhesive resin cements. These cements are able to interact with hydroxyapatite through their highly acidic methacrylate monomers and therefore do not require pre-treatment of tooth surface with bonding agents.^9^ Self-adhesive cements have become popular due to their low technique sensitivity. However, their value for bonding indirect adhesive restorations without resistance form is rather limited, as their superficial interaction with tooth tissues results in lower bond strengths to enamel (even with selective etching)^10^ and dentine^11^ compared to conventional resin cements, especially in the long term.

Finally, immediate dentine sealing (IDS),^12^ also known as dual bonding,^13^ is a relatively new approach for indirect adhesive restorations that has been associated with promising clinical results for adhesive inlays, onlays^14^ and veneers.^15^ IDS relies on the dentine bonding procedure being completed before impression taking.^12^ The bond is then allowed to mature while the indirect restoration is manufactured. When the time comes to adhesively cement the indirect restoration, the resin bond is reactivated, normally by air-particle abrasion (APA), and a combination of resin adhesive and resin cement is used to complete the cementation procedure.^16^

## Factors to consider for selecting a resin cement

Other than adhesive strategy, there are a number of other factors that should be considered when selecting a resin cement (Table 2).

**Table 2:** Factors to consider for selecting a resin cement

Factors	Main options
Polymerisation mechanism	Light-cure Dual-cure Self-cure
Compatibility with bonding agent	Bespoke self-cure initiated bonding agent Compatible universal bonding agent Light-cure adhesive resin (IDS)
Chemically active luting	Chemically active cement Compatible chemically active primer
Shade	According to clinical situation
Type of luting material	Resin cement Heated light-cure composite

### Polymerisation mechanism

Modern resin lutes are mainly light- or dual-cured. Light-cure cements provide better colour stability^27^ and working time but should be avoided when the thickness (>3 mm) or opacity of the restoration makes light penetration insufficient.^28^ All resin cements should have their final cure under a glycerine-based gel (covering the margins of indirect restoration) to prevent an oxygen-inhibition layer forming.^29^

### Compatibility with bonding agent

Bonding systems for direct restorations are normally designed to be cured before restoration placement. Contrastingly, in the indirect restoration scenario, this is thought to introduce an unacceptable misfit^12^ because the bond, if cured, pools with a variable thickness of up to 500 μm.^30^ Manufacturers have therefore traditionally produced a bespoke indirect bonding agent (designed to work with their resin cements) that is capable of self-curing beneath the seated restoration. It is essential that resin cements are only used in conjunction with their recommended bonding agent as any incompatibility may impact on polymerisation. This is particularly reported for one-step self-etch adhesives, whose acidic monomers can deactivate the amine catalyst of an incompatible dual-cure resin cement.^31^

In recent years, manufacturers have sought to make indirect and direct bonding systems more interchangeable and there has been an increase in multi-mode (universal) bonding systems that can be used for either task. Some of these systems recommend the multi-mode bonding agent is cured before cementation (for example, Adhese Universal and Variolink Esthetic, Ivoclar Vivadent),^32^ where others (for example, Scotchbond Universal and RelyX Ultimate, 3M ESPE) rely on a 'dark cure' activator contained within the resin cement to cure the adhesive.^33^

### Chemically active cements

Resin cements can be classified as chemically active if they have the ability to chemically bond to restorative materials. The classic example of this is Panavia Ex (Kuraray Noritake Dental) which was released in 1983. Panavia Ex contains the monomer 10-methacryloyloxydecyl dihydrogen phosphate (MDP) that ionically adheres to the metal oxides of non-precious alloys^34^ and polycrystalline ceramics.^35^ This results in an adhesive cement that can bond to these restorative materials without the need to prime the fitting surface.

MDP was also incorporated into the subsequent generation of Panavia F2.0, but interestingly was withdrawn from the newest formulation (Panavia V5). Clinicians should be mindful when using this newest variation of Panavia that it must be combined with a dedicated MDP-containing primer but, as long as this is done, comparable clinical outcomes can be achieved.^36^

### Shade

Most resin cements come in a variety of different shades and clinicians should choose a resin cement with an appropriate shade for their application. A good example is the recommendation to use an opaque resin cement when bonding a metal resin-bonded bridge to avoid the shine through of the material.^37^ A ceramic onlay on the other hand may be better suited to a tooth-coloured cement to help hide the transition from tooth to ceramic.

### Heated composite

Pre-heating of light-cure packable composite at about 60 °C reduces its viscosity so that it can be used as a luting material.^38^ This material has been advocated because of the perceived ease of removing excess and the higher filler content compared to traditional resin cements.^39^ Heated composite has been described as the luting material for IDS in combination with posterior indirect composite^14^ or glass-ceramic restorations.^39^

Clinicians should be careful though as the decrease in viscosity after heating is very transient;^40^ thus, the restoration should be seated with meticulous pressure (plastic-coated ultrasonic tips can be useful).^39^ Although heating is reported to increase the conversion rate of the composite,^40^ the rapid temperature drop after removal from the heater^40^ makes extended light curing (60 seconds per surface) necessary to ensure full polymerisation.^39^

## Restorative materials for adhesive bonding

As well as deciding upon a bonding strategy and resin cement, the clinician must also consider how best to prepare the restoration fitting surface of the material selected.

### Glass ceramics

Feldspathic porcelain, leucite-reinforced and lithium disilicate-reinforced glass ceramics can all be prepared for resin bonding in a similar way. Hydrofluoric acid (HF) etching is followed by a silane coupling agent to prepare the surface for the resin cement.^41^

HF etching selectively dissolves the glass matrix to increase surface roughness and provide micromechanical retention for the resin cement.^41^ Concentration of 5% for 20 seconds is generally recommended for lithium disilicate and leucite-reinforced glass ceramics,^42^ while increased time (60 seconds) and concentration (10%) are advantageous for feldspathic.^43^

The silane coupling forms siloxane bonds with the exposed silica particles of the pre-etched ceramic and double carbon bonds with the organic matrix of the subsequent resin cement.^44^ The stability of silane added in a universal primer may be negatively affected by the combined acidic monomers (for example, MDP);^45^ thus, surface pre-treatment with a sole-silane primer (alone or in combination with the universal primer) has been recommended to improve bond strengths.^46^ Additionally, clinicians should consider using the proprietary silane that is recommended by the resin lute manufacturer to avoid unwanted interactions.^47^

### Indirect composite

Indirect composites can be classified into three distinct varieties:^48^

1. Conventional handmade indirect composites
2. Prefabricated CAD/CAM nanocomposite blocks (for example, Lava Ultimate, 3M ESPE)
3. Polymer-infiltrated ceramic network materials (PICNs), commercially known as 'hybrid ceramics', which are also manufactured via CAD/CAM technology (for example, Enamic, VITA Zahnfabrik).

As dentists, we are used to bonding composite resins predictably in direct restorations, but the situation with an indirect composite is more complicated. These restorations have already been polymerised (with high conversion rates) and there is typically little free monomer left to bond when the indirect restoration is delivered.^49^

There is still considerable debate in the literature over which is the optimal protocol to adhesively cement indirect composites^50^ and it is therefore difficult to guide the practitioner to the most reliable method. All of the below techniques have been shown to produce clinically effective *in vitro* bond strengths when used with conventional indirect composites:

1. APA (with aluminium oxide) followed by silane coupling agent (to bond to the silica-based filler and improve wetting)^50^
2. APA alone (to target bonding to the unreacted free monomers)^51^
3. Tribochemical coating (Rocatec/Cojet systems, 3M ESPE) followed by silane coupling agent.^52^

Regarding the newer CAD/CAM materials, it is clear that PICNs have to be treated differently from classic indirect composites. HF etching 5% for 60 seconds followed by silane and unfilled resin is recommended.^53^^,^^54^

Due to their relative novelty, there is still some debate around how to predictably treat nanocomposite blocks.^55^ Manufacturers and some studies are recommending APA or tribochemical coating in combination with a universal bonding agent from the same manufacturer,^23^^,^^56^ but this has been contradicted^57^ and needs independent verification with more research.

### Metal alloys

Metal alloys can be classified into non-precious (for example, nickel chromium and cobalt chromium) and precious (for example, type IV gold and palladium rich).

Non-precious alloys readily form an oxide surface layer that chemically bonds to the phosphate ester groups of MDP.^34^ Therefore, predictable adhesive bonding can be achieved by using an MDP-containing primer or cement after APA of the fitting surface.^34^

APA is used to roughen the metal surface and promote micromechanical retention of the resin cement.^58^ This should be followed by ultrasonic bath cleaning to remove loosely retained alumina particles that could reduce resin bond strength to the alloy.^58^

Precious alloys do not provide a convenient oxide layer compatible with MDP bonding and thus have different bonding considerations. There is no consensus in the literature on a single method of precious alloy bonding that is preferable. Instead, all of the following methods have been tested and shown to produce a clinically acceptable bond strength to resin:

1. Heat treating the metal in the laboratory (to force a copper oxide layer to form) followed by MDP^59^
2. Tribochemical coating followed by silane coupling agent^60^
3. APA followed by a primer containing specific sulphate monomers that chemically adhere to the precious metal surface.^61^

Manufacturers have been quick to combine sulphate monomers with MDP into single-bottle metal primers. This has simplified the alloy bonding process considerably as it has meant that the same steps can be used for either class of material, although the benefit of these combined primers for non-precious alloys (compared to MDP only) is questionable.^62^

### Polycrystalline ceramics

Aluminium oxide (alumina) ceramics (for example, glass-infiltrated alumina, densely-sintered high-purity alumina) and zirconia (for example, 'high-strength' yttria-stabilised tetragonal, 'high-translucency' cubic-phase-containing) are all classed as polycrystalline ceramics.

Adhesive bonding of polycrystalline ceramics has been the subject of considerable debate in the dental literature, given that, unlike glass ceramics, these are not suitable for HF etching due to the lack of silica from their surface.^63^ Among many different bonding protocols that have been attempted, the body of evidence from laboratory and clinical data suggests that APA with 50 μm aluminium oxide followed by an MDP-containing primer or resin cement is the preferable surface treatment for zirconia.^64^^,^^65^ Tribochemical coating followed by silane is an alternative technique that gives predictable bond with alumina ceramics but should be avoided for zirconia as the silica layer on the surface appears to be unstable over time.^64^

APA should be performed at a moderate pressure of 2.5 bars to maintain the balance between possible surface damage and sufficient bond strength,^66^ although lower pressure or particle size may be employed for the weaker cubic-phase-containing zirconia.^67^

## Managing contaminants

### Restoration fitting surface

After leaving the laboratory, restorations are frequently tried in on both the working cast and in the mouth, to check fit and aesthetics before definitive cementation. This opens up the possibility of contamination with gypsum,^68^ blood,^69^ saliva,^70^ silicone fit checkers^71^ and try-in pastes,^72^ all of which have been shown to negatively affect bonds strengths.

In an ideal world, all fitting surface treatment would occur immediately after the try-in procedures, but in reality, this is seldom possible. Dental practices are often not equipped with the steam cleaners, air abrasion and ultrasonic bath equipment recommended for surface treatment. In addition, HF is a hazardous chemical which dentists may understandably prefer not to store or use in the dental practice setting.^73^ Consequently, clinicians and laboratories must be cognisant of the potential contaminants that can occur before cementation and work together to optimise fitting surface preparation.

#### Glass ceramics and indirect composites

The HF surface treatment of glass ceramics is often completed by the dental laboratory and is usually followed by immersion in a neutralising solution (to avoid over-etching) and ultrasonic bath cleaning.^74^ Saliva contamination after this procedure will result in reduced bond strengths unless it is mitigated for.^71^

A simple way to do this is to apply the silane before intraoral try-in, as this appears to help the ceramic resist saliva contamination and restore the bond strength of the resin cement.^68^ Alternatively, phosphoric acid (30 seconds) or a proprietary cleaning paste can be applied to the fitting surface following try-in and before applying the silane to similar effect.^75^ Cleaning the restoration with phosphoric acid has the additional benefit of removing glass precipitates that collect following HF etching, which is also thought to improve bond strengths.^74^

Unfortunately, silicone and try-in paste contamination is more difficult to remove from glass ceramics^71^^,^^72^ and, if their use is planned, dentists may wish to defer HF etching until after the try-in procedures have been completed.^39^

If an indirect composite restoration has APA completed before try-in procedures, then phosphoric acid etching appears to be similarly effective at removing saliva contamination before applying either silane or adhesive resin.^69^ For PICNs, post-HF etching cleaning with phosphoric acid does not appear to be beneficial, unlike glass ceramics.^54^

#### Polycrystalline ceramics/non-precious alloys/heat-treated precious alloys

Both polycrystalline ceramics and metal alloys will become contaminated with saliva upon intraoral try-in, resulting in decreased bond strengths.^70^ Saliva contamination can be removed by steam cleaning and re-sandblasting the intaglio at a pressure of 2.5 bars for 15 seconds.^70^

When APA is unavailable, dentists are also able to remove saliva contaminants by applying a proprietary zirconium-based cleaning paste (Ivoclean, Ivoclar Vivadent) or cleaning with sodium hypochlorite.^76^

It is important that clinicians do notattempt toclean polycrystalline ceramics or metal alloys with phosphoric acid at any time. This is because the phosphate will chemically adhere to the metal oxide surface layer, which in turn will leave no free binding sites for the MDP, resulting in diminished bond strengths.^70^

### Tooth substrate

**Fig. 3:**
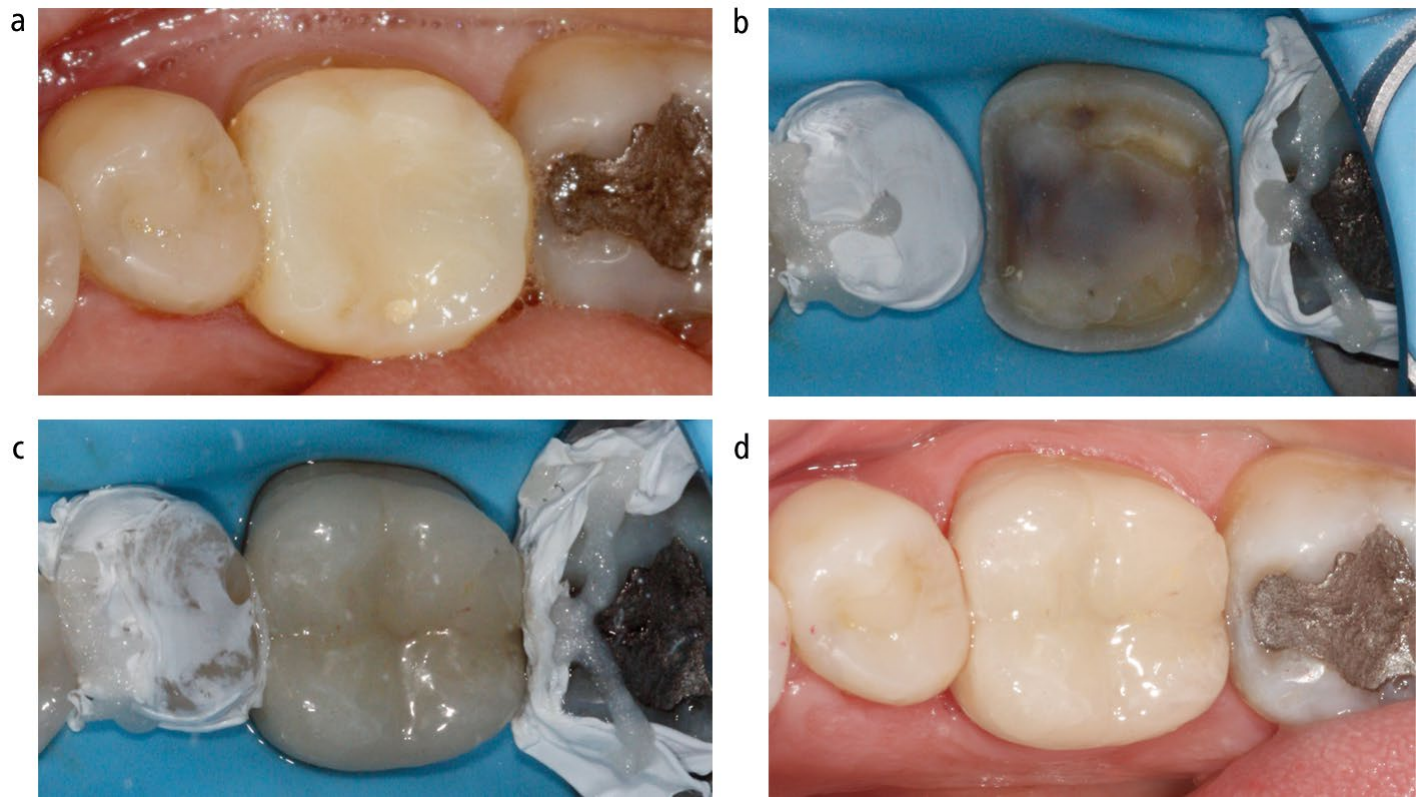
Cementation process for the tooth prepared in Fig. 2 The provisional restoration (a) is first removed, then the tooth is isolated and intraoral APA is completed (b), before bonding the restoration (c). d) Shows the adhesively cemented lithium disilicate restoration at two-year recall

As previously discussed, the adhesive cementation procedure is especially susceptible to moisture contamination,^5^ so isolation (ideally with a rubber dam) should always be considered when performing adhesive cementation (Fig. 3).

In addition, dentists should be mindful that resin cement polymerisation may be negatively affected by eugenol-based temporary cements,^77^ haemostatic agents^78^ and oxidative solutions (especially up to three weeks post-bleaching).^79^

Finally, intraoral APA of the tooth substrate should be employed whenever possible before adhesive cementation (Fig. 3b) as it removes biofilm, stains and temporary cement residues,^80^ while also having a positive effect on bond strength to dentine,^81^ enamel (particularly when employing a self-etching strategy),^22^ existing composite restorations^82^ and previously performed IDS.^16^

## Conclusion

There are many factors that can influence the predictability of the bond achieved when adhesively cementing indirect restorations. This article provides a summary of these factors to help dentists and dental laboratories plan their adhesive protocols and maximise the success of their adhesive indirect restorations.
